# Supplementary material for: Plitidepsin: Mechanisms and Clinical Profile of a Promising Antiviral Agent against COVID-19
Source: J Pers Med. 2021 Jul 16;11(7):668. doi: 10.3390/jpm11070668 (PMC8306251; doi:10.3390/jpm11070668)
Supplement: Supplementary file 1 [file jpm-11-00668-s001.zip › jpm-1226006-supplementary.pdf]

Supplementary S1. Search strategy.

1.Initial search (date: March 23, 2021).

<u>A. Plitidepsin-oriented search</u>

A1. PubMed (164 results)

(plitidepsin OR "plitidepsin" [Supplementary Concept] OR aplidine OR aplidin OR "dehydrodidemnin B")

A2. Scopus (416 results)

(plitidepsin OR aplidine OR aplidin OR "dehydrodidemnin B")

A3. CENTRAL (14 results)

(plitidepsin OR aplidine OR aplidin OR "dehydrodidemnin B")

A4.clinicaltrials.gov (13 results)

Condition or disease: (-); Other terms: plitidepsin; Country: (-)

<u>B. SARS-CoV-2-oriented search</u>

A1. PubMed (6 results)

(plitidepsin OR "plitidepsin" [Supplementary Concept] OR aplidine OR aplidin OR "dehydrodidemnin B") AND ("COVID-19"[Mesh] OR COVID-19 OR “Coronavirus disease 19” OR "SARS-CoV-2"[Mesh] OR SARS-CoV-2 OR “severe acute respiratory syndrome coronavirus 2”)

A2. Scopus (2 results)

(plitidepsin OR aplidine OR aplidin OR "dehydrodidemnin B") AND (COVID-19 OR “Coronavirus disease 19” OR SARS-CoV-2 OR “severe acute respiratory syndrome coronavirus 2”)

A3. CENTRAL (1 result)

(plitidepsin OR aplidine OR aplidin OR "dehydrodidemnin B") AND (COVID-19 OR “Coronavirus disease 19” OR SARS-CoV-2 OR “severe acute respiratory syndrome coronavirus 2”)

A4.clinicaltrials.gov (2 results)

Condition or disease: COVID-19; Other terms: plitidepsin; Country: (-)

A5.medRxiv&bioRxiv (1 relevant paper acquired)

(plitidepsin OR aplidine OR aplidin OR "dehydrodidemnin B") AND (COVID-19 OR “Coronavirus disease 19” OR SARS-CoV-2 OR “severe acute respiratory syndrome coronavirus 2”)

2. Update of search(last search date:April 15, 2021)

A. Plitidepsin-oriented search

A1. PubMed (1 new result)

(plitidepsin OR "plitidepsin" [Supplementary Concept] OR aplidine OR aplidin OR "dehydrodidemnin B")

A2. Scopus (3new results)

(plitidepsin OR aplidine OR aplidin OR "dehydrodidemnin B")

A3. CENTRAL (1 new result)

(plitidepsin OR aplidine OR aplidin OR "dehydrodidemnin B")

A4.clinicaltrials.gov (0 new results)

Condition or disease: (-); Other terms: plitidepsin; Country: (-)

B. SARS-CoV-2-oriented search

A1. PubMed (1 new result)

(plitidepsin OR "plitidepsin" [Supplementary Concept] OR aplidine OR aplidin OR "dehydrodidemnin B") AND ("COVID-19"[Mesh] OR COVID-19 OR "Coronavirus disease 19" OR "SARS-CoV-2"[Mesh] OR SARS-CoV-2 OR "severe acute respiratory syndrome coronavirus 2")

A2. Scopus (4 new results)

(plitidepsin OR aplidine OR aplidin OR "dehydrodidemnin B") AND (COVID-19 OR "Coronavirus disease 19" OR SARS-CoV-2 OR "severe acute respiratory syndrome coronavirus 2")

A3. CENTRAL (1 new result)

(plitidepsin OR aplidine OR aplidin OR "dehydrodidemnin B") AND (COVID-19 OR "Coronavirus disease 19" OR SARS-CoV-2 OR "severe acute respiratory syndrome coronavirus 2")

A4.clinicaltrials.gov (0new results)

Condition or disease: COVID-19; Other terms: plitidepsin; Country: (-)
